# Extraction, purification and *in vitro* assessment of the antioxidant and anti-inflammatory activity of policosanols from non-psychoactive *Cannabis sativa* L.

**DOI:** 10.1016/j.heliyon.2024.e30291

**Published:** 2024-04-26

**Authors:** Clarissa Caroli, Giovanna Baron, Giorgio Cappellucci, Virginia Brighenti, Larissa Della Vedova, Francesca Fraulini, Simonetta Oliaro-Bosso, Andrea Alessandrini, Alfonso Zambon, Gigliola Lusvardi, Giancarlo Aldini, Marco Biagi, Lorenzo Corsi, Federica Pellati

**Affiliations:** a Department of Life Sciences, University of Modena and Reggio Emilia, Via Giuseppe Campi 103-287, 41125, Modena, Italy; b Clinical and Experimental Medicine PhD Program, University of Modena and Reggio, Via Giuseppe Campi 287, 41125, Modena, Italy; c Department of Pharmaceutical Sciences, University of Milan, Via Mangiagalli 25, 20133, Milan, Italy; d Department of Physical Sciences, Earth and Environment, University of Siena, Via Laterina, 8, 53100, Siena, Italy; e Department of Chemical and Geological Sciences, University of Modena and Reggio Emilia, Via Giuseppe Campi 103, 41125, Modena, Italy; f Department of Drug Science and Technology, University of Turin, Via Pietro Giuria 9, 10125, Turin, Italy; g Department of Physics, Informatics and Mathematics, University of Modena and Reggio Emilia, Via Giuseppe Campi 213/A, 41125, Modena, Italy; h National Institute of Biostructures e Biosystems (INBB), 00136, Roma, Italy; i Department of Food and Drug, University of Parma, Parco Area delle Scienze 27/A, 43124, Parma, Italy

**Keywords:** *Cannabis sativa* L., Policosanols, Extraction, HPLC, Antioxidant activity, Anti-inflammatory activity

## Abstract

Policosanols (PCs) are bioactive compounds extracted from different natural waxes. In this work, the purification, characterization and assessment of the antioxidant and anti-inflammatory activity was carried out on PCs from an innovative source, i.e. a waxy material from supercritical-fluid extraction (SFE) of non-psychoactive *Cannabis sativa* L. (hemp) inflorescences. Starting from this material, PCs were obtained by microwave-assisted *trans*-esterification and hydrolysis, followed by preparative liquid chromatography under normal phase conditions. The purified product was characterized using high-performance liquid chromatography (HPLC) with an evaporative light scattering detector (ELSD). *In vitro* cell-free and cell-based antioxidant and anti-inflammatory assays were then performed to assess their bioactivity.

HPLC-ELSED analysis of the purified mixture from hemp wax revealed C_26_OH and C_28_OH as the main compounds. *In vitro* assays indicated an inhibition of intracellular reactive oxygen species (ROS) production, a reduction of nuclear factor kappa B (NF-κB) activation and of the activity of the neutrophil elastase. Immunoblotting assays allowed us to hypothesize the mechanism of action of the compounds of interest, given the higher levels of MAPK-activated protein kinase 2 (MK2) and heme oxygenase-1 (HO-1) protein expression in the PC pretreated HaCaT cells. In conclusion, even if more research is needed to unveil other molecular mechanisms involved in hemp PC activity, the results of this work suggest that these compounds may have potential for use in oxinﬂammation processes.

## Highlights

• A new extraction method for policosanols from hemp wax was developed.
• The purified mixture was fully characterized using HPLC-ELSD.
• Hemp PCs inhibit ROS production and modulate MK2 and HO-1 expression.
• Hemp PCs reduce NF-κB activation and the activity of the neutrophil elastase.
• This new product may be useful against oxinﬂammation processes.

## Graphical abstract

**Figure:**
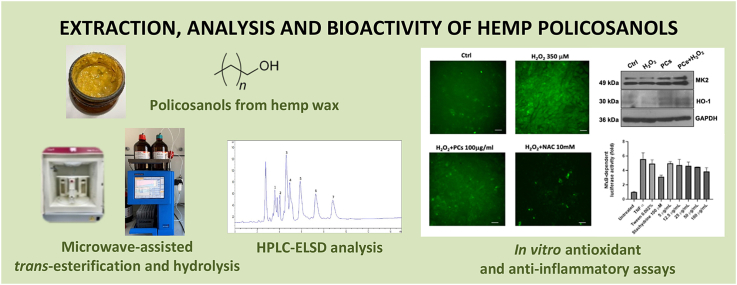

## 1 Introduction

Policosanols (PCs) are a mixture of long chain aliphatic primary alcohols (C_20_OH–C_36_OH). Sugar cane represents the main starting material to extract these compounds [1]. However, they have been identified in many other plant and animal waxes, including *Cannabis sativa* L. inflorescences [2,3].

PCs have been the focus of many studies from the chemical point of view and they have been mainly used in food supplements to decrease cholesterol levels [[3], [4], [5]], even if this effect is still controversial [6]. As far as the possible properties of PCs against oxidative stress, inflammation and cancer, available data are limited [5]. *In vitro* assays have been performed in previous studies to evaluate the antioxidant activity of PCs [7,8]. More recently, Cho et al. have described the capacity of a PC mixture to prevent LDL-oxidation as well as to possess an anti-inflammatory activity, being them able to protect zebrafish embryo death in the presence of carboxymethyl-lysine [9]. Recent studies have demonstrated the role of PCs from insect waxes as effective products on *Caenorhabditis elegans* models of both Parkinson's and Alzheimer's diseases [10,11]. As for the antiproliferative activity, PCs have been investigated *in vitro* against human gastric SNU-16 cancer cells and in an *in vivo* xenograft mouse mode [12].

The main limitations of existing methods for the extraction of PCs from natural sources are a long extraction time and a low recovery efficiency [5]. PCs are indeed present in very low amount in raw waxy material since they are bonded to fatty acids to form esters. For this reason, it is necessary to perform either a saponification or a *trans*-esterification procedure to free them from their ester form [5]. A microwave-assisted method has been optimized for the extraction of PCs from beeswax in a shorter time and with a good yield in comparison with conventional methods [13]. PCs from beeswax are mainly long-chain alcohols, including C_30_OH and C_32_OH [13]. Regarding PCs from *C. sativa*, compounds from C_24_ to C_32_ are the most abundant ones [2,3]. Brighenti et al. have analyzed several varieties of non-psychoactive *C. sativa* (hemp) inflorescences, highlighting a different profile of PCs in the samples [2]. PCs with odd chain length have also been detected in a low amount in hemp [3].

In the light of this, this research was aimed at the extraction and purification of PCs from hemp wax, using a microwave-assisted *trans*-esterification and hydrolysis reaction combined in a single step. The purification of the raw extract was carried out by preparative flash column chromatography under normal-phase (NP) conditions. The composition of the purified mixture of PCs was assessed using a fully validated method based on high-performance liquid-chromatography (HPLC) under reversed-phase (RP) conditions with an evaporative light scattering detector (ELSD), which represents a very suitable tool for the analysis of these compounds in hemp extracts [2]. The antioxidant and anti-inflammatory activity of PCs from hemp wax was assessed *in vitro* to evaluate their application in both the pharmaceutical and nutraceutical fields.

## 2 Materials and methods

### 2.1 Chemicals and solvents

*n*-Hexane, ethanol (EtOH), chloroform (CHCl_3_), dichloromethane (DCM), acetonitrile (ACN) and methanol (MeOH), all HPLC purity grade (≥99.8 %), sodium and potassium hydroxide (NaOH and KOH), Tris-HCl, sodium chloride (NaCl), sodium deoxycolate, hydrogen peroxide (H_2_O_2_), N-acetylcysteine (NAC), Triton X-100, phenylmethylsulfonyl fluoride (PMSF) and phosphate buffered saline (PBS) were purchased from Sigma-Aldrich (Milan, Italy). Reference compounds, including C_24_OH (≥99 %), C_26_OH (≥97 %), C_28_OH (≥99 %) and C_30_OH (≥96 %), were from Sigma-Aldrich (Milan, Italy). 3-(4,5-Dimethyl-2-thiazolyl)-2,5-diphenyl-2H-tetrazolium bromide (MTT), hydrochloric acid (HCl), dimethyl sulfoxide (DMSO), Tween20 and human tumor necrosis factor-α (TNFα) were from Merck KGaA, Darmstadt, Germany. Stachydrine was purchased from Biosynth Carbosynth (Compton, Newbury, United Kingdom). HPLC purity grade methyl methyl *tert*-butyl ether (MTBE) was from Carlo Erba (Milan, Italy). Water (H_2_O) was purified by using a 185 Millipore Milli-Q Plus System (Milford, MA, USA). Dulbecco's Modified Eagle Medium (DMEM), DMEM/F12, fetal bovine serum (FBS), l-glutamine and penicillin-streptomycin (Pen-Strep) were purchased from Euroclone (Milan, Italy).

### 2.2 Raw plant material

The wax material obtained by supercritical fluid extraction (SFE) with carbon dioxide (CO_2_) of non-psychoactive *C. sativa* inflorescences (hemp, Kompolti variety) was provided by Exenia s.r.l. (Pinerolo, TO, Italy).

### 2.3 Isolation of PCs from hemp wax

#### 2.3.1 Microwave-Assisted Extraction (MAE) of PCs

The microwave-assisted procedure to obtain free PCs from hemp wax was developed from a previous study carried out on beeswax [13]. In particular, the *trans*-esterification of the wax esters and the hydrolysis were condensed in one-step assisted by the microwave technology. A portion of 10 g of the waxy material was weighed and divided into two closed microwave vessels and added with 20 mL of EtOH, 20 mL of 2 M NaOH and 200 mg of solid NaOH. The vessels were then placed in the microwave apparatus (FlexiWAVE, Milestone, Sorisole (BG), Italy), where the temperature and the holding time were set at 120 °C and 15 min, respectively, with a medium speed magnetic stirring. The dark, red-colored smooth product obtained was vacuum filtered and washed with approximately 800 mL of H_2_O. Lastly, the raw product was let to dry completely in an oven at 35 °C for one day and in a desiccator overnight.

#### 2.3.2 Purification of PCs by preparative liquid chromatography

The raw product was submitted to a further purification by means of preparative flash column liquid chromatography with an Isolera™ One flash purification system (Biotage AB, Uppsala, Sweden). The separation of PCs from other lipophilic compounds was performed by loading 1 g of the mixture on a silica gel SNAP cartridge (25 g) (Biotage AB, Uppala, Sweden), under isocratic elution with DCM at a flow rate of 25 mL/min. The volume of the fractions collected was 18 mL. The elution was monitored by thin layer chromatography (TLC), together with a standard mixture of alkanes (from C_8_ to C_40_) and policosanols (C_24_OH, C_26_OH, C_28_OH, C_30_OH), with a mobile phase consisting of DCM-CHCl_3_ (1:1 v/v). For the visualization of the bands, plates were dipped in a cerium sulphate solution and, subsequently, heated until the blue-colored spots were visible. Fractions containing the compounds of interest were then pooled and brought to dryness under vacuum.

The purified mixture of PCs was finally submitted to HPLC-ELSD. For this purpose, 7 mg of sample was dissolved in 1 mL of CHCl_3_ and filtered through syringe 0.22 μm PTFE filter before injection into the HPLC system.

### 2.4 HPLC-ELSD analysis of PCs

The HPLC-ELSD analyses of PCs in the purified mixture from hemp wax were performed on an Agilent Technologies (Waldbronn, Germany) modular model 1260 Infinity II system, with a vacuum degasser, a quaternary pump and a thermostated column compartment [2]. The separation of the compounds of interest was carried out on an Atlantis™ dC_18_ column (150 × 3.0 mm, 3 μm, Waters, Milford, MA, USA). The mobile phase was composed of ACN (solvent A) and a mixture MTBE-MeOH 90:10 (v*/*v) (solvent B), under the following gradient: 0–1 min isocratic elution at 20% B; 1–16 min linear gradient from 20 to 45% B, which was held constant for 4 min; the column was finally brought to 20% B in 5 min. The flow-rate was 1.5 mL/min and the injection volume was 10 μL. The ELSD evaporator temperature was set at 35 °C, while nebulizer temperature was 30 °C. Nitrogen flow rate was set at 1.50 SLM. All the samples analyzed in this study were injected in duplicate.

### 2.5 Sample preparation for biological assays

To favor the solubilization of the PC purified mixture in the medium, a mixture of EtOH-DMSO (50:50, v/v) was used; then, Tween20 was added to reach a final concentration of 0.002%. The stock solution (20 mg/mL) was diluted with the cell culture medium to get the desired concentration range.

### 2.6 Cell culture and cell treatment

HaCaT keratinocytes were obtained from the American Type Culture Collection (Manassas, VA, USA) and cultured in DMEM/F12 with 10% heat-inactivated FBS, l-glutamine 1% and Pen-Strep 1%. Cells were kept in a humidiﬁed incubator at 5% CO_2_ and 37 °C until 80% of cell confluence. Once the confluence was reached, cells were treated with PCs (5–100 μg/mL) for 12 h and, subsequently, with 350 μM of H_2_O_2_ for 30 min. All the tests have been performed in serum-free medium.

### 2.7 Determination of reactive oxygen species content in HaCaT cells

The intracellular reactive oxygen species (ROS) level was evaluated using a commercial H_2_DCF-DA kit (Abcam ab133851), containing a chloromethyl derivative of 2′,7′-dichlorodihydrofluorescein diacetate, which is an oxidative stress indicator. In the presence of ROS inside cells, H_2_DCF-DA is converted into dichlorofluorescein (DCF), a fluorescent adduct. Therefore, the fluorescence signal intensity of DCF is proportional to the amount of ROS produced by the cells.

To test the capacity of PCs to reduce ROS intracellular level in H_2_O_2_ stimulated keratinocytes, HaCaT cells were seeded in 96-well plates at a cell density of 2.5 × 10^4^ cells/well and cultured for 16 h before the experiment. Then, cells were treated with PCs (5, 12.5, 25, 50, 100 μg/mL) for 12 h. After this, the medium was removed and cells were rapidly washed with the buffer. Then, cells were stimulated for 20 min with 350 μM H_2_O_2_. After the stimulation, 100 μL of the diluted DCFDA solution was added to each well and plates were incubated for 45 min at 37 °C in the dark. Subsequently, the DCFDA solution was removed and 100 μL of buffer were added. NAC 10 mM was used as the positive control. Using the microplate reader VICTOR® Nivo™ 3s, (PerkinElmer Italia, Milan), fluorescence was recorded at λ_Ex/Em_ 488/535 nm, every 5 min for 1 h. All samples were analyzed in replicate (*n* = 6). ROS level produced by tested samples was reported as fold change in comparison with not treated cells (control), normalized as 1.

To examine the effect of the PC treatment on intracellular ROS production in H_2_O_2_-stimulated HaCaT cell line in different and integrative way, the fluorogenic probe CellROX® Green Flow Cytometry Assay Kit (Life Technologies, Milan, Italy) was used. Briefly, HaCaT derived keratinocytes cells were plated in μ-slide-8 well glass bottom (Ibidi, USA) at 6 × 10^4^ cells/well and kept in incubator overnight. Cells were then stimulated for 30 min with 350 μM H_2_O_2_ in the absence or presence of PC pre-treatment (100 μg/mL) or NAC (10 mM) (Sigma Aldrich. Milan). Subsequently, 10 μL of “CellROX Green” solution were added in each well 30 min before the end of the treatment at the concentration of 5 μM and ﬂuorescence was measured by a 20 × ﬂuorescence microscope Olympus IX 70 inverted microscope equipped with a Digital Sight camera DS-Qi2 (Olympus Life Science, Tokyo, Japan). Quantitative analysis was carried out using ImageJ (NIH) version 1.54e (USA) software. The results were expressed as a percentage of the control group.

### 2.8 Assessment of the antioxidant activity against catalase and superoxide dismutase

The antioxidant activity of the purified mixture of PCs was assessed by means of enzymatic assays based on their ability to remove H_2_O_2_ and the radical superoxide anion O_2_^•–^, which represent two of the most significant ROS species. In analogy with the role of the enzyme catalase (CAT) and superoxide dismutase (SOD), these were named CAT-like activity and SOD-like activity, respectively [14,15]. For each assay the sample was tested at 5, 12.5, 25, 50 and 100 μg/mL and NAC at 10 and 1 mM was used as a reference.

CAT-like activity tests were performed using the Fluorimetric Hydrogen Peroxide Assay Kit (Sigma-Aldrich) with a TECAN GeniosPro microplate reader. The presence of H_2_O_2_ was detected thanks to its reaction with a molecular probe catalyzed by the peroxidase enzyme, which generates a red fluorescent product that can be analyzed fluorometrically. CAT activity is reported as the percentage of H_2_O_2_ decomposed at the end of the assay. Twenty μL of each sample was treated with 200 μL of a 50 μM solution of H_2_O_2_ in buffer solution and measured the residual H_2_O_2_ concentration after 30, 60 and 120 min. Two replicates were performed for each sample.

SOD-like activity tests were carried out using the SOD determination kit (Sigma-Aldrich), adapted for a UV–Vis spectrophotometer (JASCO V-570). In this assay, the SOD-like activity is expressed as the inhibition rate (I.R.%) of the formation of a H_2_O-soluble formazan dye, generated upon reduction of a tetrazolium salt (WST-1) by the superoxide anion catalyzed by xanthine oxidase and inhibited by SOD. This activity was evaluated after 20 min of treatment at 440 nm.

### 2.9 Immunoblotting

Western blot was used to measure the expression of MAPK-activated protein kinase 2 (MK2) and heme oxygenase-1 (HO-1) in H_2_O_2_ induced ROS production in HaCaT cells pre-treated or not with PCs. Brieﬂy, 350 μM H_2_O_2_ and PC (100 μg/mL) treated HaCaT cells were lysed and extracted in RIPA buffer (50 mM Tris-HCl pH 7.4, 150 mM NaCl, 1% sodium deoxycolate, 1% Triton X-100, 2 mM PMSF). Protein quantification was carried out using BCA Protein Assay Kit (Life Technology, Milano, Italy), following the manufacturer instruction. Equal amounts of proteins (5 μg for each sample) were separated through 5–12% SDS-PAGE mini gel and transferred onto nitrocellulose membrane (Life Technology, Milano, Italy). The membranes were then incubated with primary rabbit antibody anti-MK2 (1:2000) (Cell Signaling Technology, USA) and anti–HO–1 (1:1000) (Abcam, UK) overnight at 4 °C. Then, the membranes were washed three times in TBST, incubated for 1 h with HRP-conjugated anti-rabbit antibody (Thermofisher, USA), and protein bands were observed and photographed. The densitometry analyses were performed using the Imagestudio lite software, with glyceraldehyde 3-phosphate dehydrogenase (GAPDH) as the loading control.

### 2.10 Evaluation of the anti-inflammatory activity of the purified mixture and cellular vitality

The *in vitro* anti-inflammatory activity of PCs from hemp wax was assessed using a validated cellular model previously described [16], where the inflammatory target nuclear factor kappa B (NF-κB) is monitored using a gene-reporting method in commercial R3/1-NF-κB cells. To verify that the presence of Tween20 did not affect the anti-inflammatory activity of the hemp wax, Tween20 at its highest concentration was also tested.

Briefly, R3/1-NF-κB cells were seeded (4000 cells/well) in a white 96-well plate (BRANDplates®, cell grade). Cells were pre-treated with different concentrations of hemp PCs (5–100 μg/mL) for 18 h in complete medium (DMEM 10% FBS, 1% l-glutamine, 1% Pen-Strep), followed by a 6 h stimulation with 10 ng/mL TNFα. Stachydrine (100 μM) was used as the positive control. To avoid component interference with the reading of the luciferase assay, cells were washed once with 100 μL of warm PBS and 50 μL of DMEM was then added. Next, 50 μL ONE-Glo™ Luciferase Assay Substrate (Promega Corporation, Madison, WI, USA) was directly added to the wells, followed by a luciferase measurement performed using a luminometer (Wallac Victor2 1420, PerkinElmer™ Life Science, Monza, Italy).

The MTT assay was applied to assess cell viability for the all the concentrations tested in the anti-inflammatory assay. After 18 h incubation with hemp PCs (5–100 μg/mL) and Tween20 (0.002 %), the MTT reagent was added and incubated for 4 h. After medium removal, cells were lysed and MTT was solubilized by adding 100 μL of lysis buffer (8 mM HCl, 5% Tween20, DMSO). The 96-well plate was shaken for 15 min and the absorbance at 575 nm was measured using a plate reader (BioTek's PowerWave HT, Winooski, VT, USA). Cells incubated with complete medium were used as a control for 100% cell proliferation.

### 2.11 Determination of the neutrophil elastase inhibitory activity

The inhibitory effect of neutrophil elastase was assessed using the Sigma-Aldrich kit Assay (Sigma-Aldrich, Milan, MAK213), which is based on the interaction between elastase and a synthetic substrate. The proteolytic cleavage releases a fluorophore which can be easily quantified by fluorescence. The test was performed according to the data sheet in the range of final concentration of tested sample 5–100 μg/mL. Enzyme activity and inhibition exerted by tested samples were calculated and IC_50_ value extrapolated from the curve concentration/inhibition activity.

### 2.12 Statistical analysis

Experiments for the assessment of the bioactivity of the PC purified mixture were performed with biological and technical replicates and the results are shown as mean ± standard error of the mean (SEM), compared to untreated control cells. Statistical analysis was performed using one-way analysis of variance (ANOVA) with Bonferroni's multiple comparisons test (*p* < 0.05 was considered significant), using GraphPad Prism 9.0.1 software (San Diego, CA, USA, www.graphpad.com, accessed on July 11, 2022).

## 3 Results and discussion

### 3.1 Extraction and purification of PCs from hemp wax

The sample of hemp wax investigated in this research work was produced by SFE with CO_2_ from hemp female inflorescences. The extraction procedure to obtain PCs followed for this matrix was developed from the one previously described by Venturelli et al. for beeswax [13]. In this case, a single step process was preferred for hemp wax, as it proved to be equally efficient as the two-step process in term of product yield, but with a significant time saving, as previously described for hemp inflorescences. The final mixture appears as an intense red colored waxy material, the color being due to the oxidation of cannabidiol (CBD) into cannabidiolquinone (CBDQ), occurring in an alkaline alcoholic environment. This fact is not surprising, being hemp wax obtained by SFE from hemp inflorescences; indeed, it contains not only PCs and fatty acids, but also neutral cannabinoids, such as CBD. Nevertheless, this compound can be easily removed, together with free fatty acid salts, by washing with H_2_O. The yield of this one-step *trans-*esterification and hydrolysis procedure was around 55% with respect to the starting material.

For the removal of non-polar impurities, such as *n*-alkanes, a purification by means of preparative liquid chromatography was then carried out on the raw product. A total of 27 fractions were collected and monitored for their qualitative profile by TLC (Fig. S1, Supplementary Information). The Rf values were found to be 0.10 for C_24_OH, 0.16 for C_26_OH, 0.22 for C_28_OH and 0.30 for C_30_OH, respectively. Fractions from 22 to 26 were pooled and brought to dryness as they were observed to contain the PCs mixture (overall yield 7.0%).

### 3.2 Qualitative and quantitative analysis of PCs in the purified mixture obtained from hemp wax

**Fig. 1:**
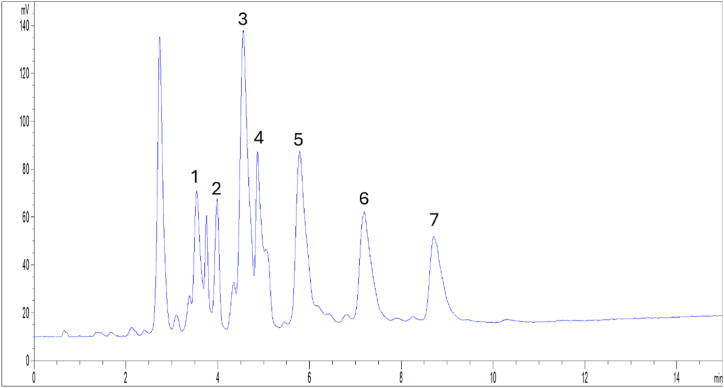
HPLC-ELSD chromatogram of the PC mixture purified from hemp wax using flash chromatography (fractions 22–26). For peak identifications see Table 1.

Quantification of PCs in the purified mixtures obtained from hemp wax was obtained by means of a validated HPLC-ELSD method [2]. Fig. 1 shows a representative HPLC-ELSD chromatogram of PCs from hemp wax. Resolution (Rs) was found to be higher than 1.50 for all peaks, except for peak 4.

Quantitative data related to the content of PCs in the product purified from hemp wax are shown in Table 1. Total PC amount wax was interestingly high with a value of 698.6 ± 9.5 mg/g, thus highlighting the role of this product in the pharmaceutical and nutraceutical fields. In the purified mixture the major compounds were found to be C_26_OH and C_28_OH. Together with PCs, other compounds are present in the final mixture and eluted at the beginning of the chromatographic run, mainly long-chain fatty acids that were not totally removed during the H_2_O-washing process, as confirmed by the analysis using gas chromatography coupled with mass spectrometry (GC-MS) [13].

**Table 1:** Content of PCs in the mixture purified from hemp wax (fractions 22–26). Data are expressed as mg/g ± SD (*n* = 4).

**Peak number**	Compound	R_t_ (min)	mg/g
**1**	C_24_OH	3.5	85.8 ± 3.9
**2**	C_25_OH	3.9	64.1 ± 3.8
**3**	C_26_OH	4.6	181.1 ± 0.2
**4**	C_27_OH	4.9	77.2 ± 1.0
**5**	C_28_OH	5.8	130.5 ± 2.0
**6**	C_30_OH	7.2	83.7 ± 1.1
**7**	C_32_OH	8.7	76.5 ± 0.1
**Total**			698.6 ± 9.5

### 3.3 Effect of hemp PCs on the production of intracellular ROS

Stress is known to exert repercussions on several aspects of cell life. Various experimental research has demonstrated how intrinsic and extrinsic (smoking, UV exposure, environmental pollution) biological mediators of stress are able to induce aging in biological matrices and they can significantly promote cellular senescence, which is an important contributor to intrinsic and extrinsic skin aging [17]. Thus, among other factors, such as inhibition of telomerase activity and mitochondrial damage, oxidative damage plays a fundamental role [18,19]. Indeed, ROS produced through H_2_O_2_ is one of the trigger points to the molecular response of skin cells. In the light of this, the effect of acute exposure of HaCaT cells to 350 μM H_2_O_2_ was assessed. The choice of the exposure time and concentration was based on dose-response experiments at different time points (Fig. S2, Supplementary Information).

**Fig. 2:**
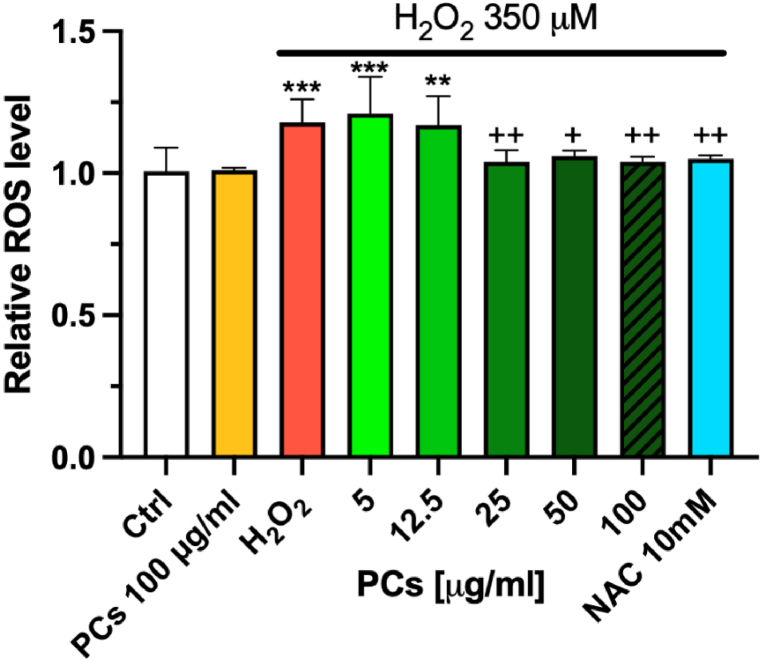
Quantification of ROS in HaCat cell line treated or not with PCs exposed to H_2_O_2_. Intracellular ROS levels were quantified after treatment with 350 μM H_2_O_2_ in HaCat cells pretreated or not with different concentrations of PCs. The ROS levels were measured from relative fluorescence intensity with H_2_DCF-DA. Data are expressed as mean ± S.D. (*n* = 7); significance one-way ANOVA with Tuckey's multiple comparison test, ***p < 0.001 and **p < 0.01 vs. Ctrl, ^++^*p* < 0.01 and+*p* < 0.05 vs H_2_O_2_).

In the first test using DCF, PCs were able to efficiently counteract intracellular ROS upregulation mediated by H_2_O_2_. In detail, DCF fluorescence markedly increased in H_2_O_2_ stimulated cells already after 10 min of exposure (+20%, *p* < 0.01 vs ctrl), but at 100, 50 and 25 μg/mL PCs inhibited ROS production in stimulated cells in a clear extent, between 67.2 ± 10.2% and 76.9 ± 10.2% (at 100 μg/mL) compared to non-treated. Differences between values obtained for different concentrations were not statistically significant, even if compared with NAC as antioxidant reference compound (Fig. 2).

**Fig. 3:**
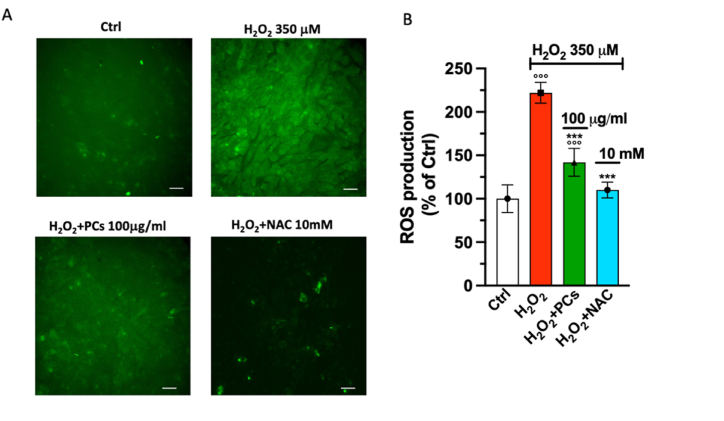
Eﬀect of PCs on H_2_O_2_ induced intracellular ROS content in HaCaT cells. (A) HaCaT cells were pretreated with PCs at 100 μg/mL for 12 h before to exposure to 350 μM H_2_O_2_ for 30 min. ROS content was detected by CellROX-Green and examined with 20 × ﬂuorescence microscope. Scale bar: 100 μm. (B) Fluorescence quantitative analysis by ImageJ. Data are expressed as mean ± S.D. (*n* = 7) and the significance one-way ANOVA with Tuckey's multiple comparison test *p* < 0.001 vs. Ctrl and ***p < 0.001 vs·H_2_O_2_ treated group.

The ROS-sensitive fluorescent dye Green CellROX was also used to optically investigate whether PCs at the most effective concentration (100 μg/mL) may prevent H_2_O_2_-induced ROS generation. HaCaT cells exposed to 350 μM H_2_O_2_ for 30 min accumulated a markedly amount of intracellular ROS, as shown by the increase of fluorescence signal in comparison to the no treated cells (Fig. 3A). On the contrary, the pretreatment with PCs dramatically reduced the intensity fluorescent green signal at the level of NAC, indicating an inhibition of intracellular ROS production (Fig. 3A). As shown in Fig. 3B, the levels of ROS production elicited by H_2_O_2_ rise to 234.9 ± 6.1%, whereas the pretreatment with PCs reduced the level of ROS to 148.0 ± 8.5%, level close to that obtained with the treatment of 10 mM of NAC (115.0 ± 4.4%).

### 3.4 Antioxidant activity of the purified mixture of PCs against CAT and SOD

The ability of PCs to reduce the H_2_O_2_-induced ROS production led us to evaluate the possible activation of protein involved in H_2_O_2_ scavenger activity. The analysis of the antioxidant activity revealed that cells treated with PCs did not show a marked antioxidant activity against H_2_O_2_ and O_2_^•–^. In fact, the CAT-like activity test showed constant H_2_O_2_ concentration up to 120 min of contact with the samples. Analogously, the SOD-like activity test did not evidence any ability of the sample to scavenge O_2_^•–^, as the samples show no significant I.R.%. In the case of NAC, we observed around 50% dismutation of H_2_O_2_ connected with the CAT-like activity, while we did not detect any SOD-like activity.

These results suggest either an alternative pathway or others antioxidant response element (ARE) involving the activity of PCs on ROS modulation. Among them, HO-1, an enzyme regulated by ARE, plays a potentially key role in antioxidant defense and iron homeostasis [20]. In addition, several research identified signal transduction pathways involved in ROS regulation, such as the MK2/Heat shock protein 27 (Hsp27) signaling [21].

### 3.5 Effect of hemp PCs on MK2 and HO-1 expression in oxidative stress-induced HaCaT cells

**Fig. 4:**
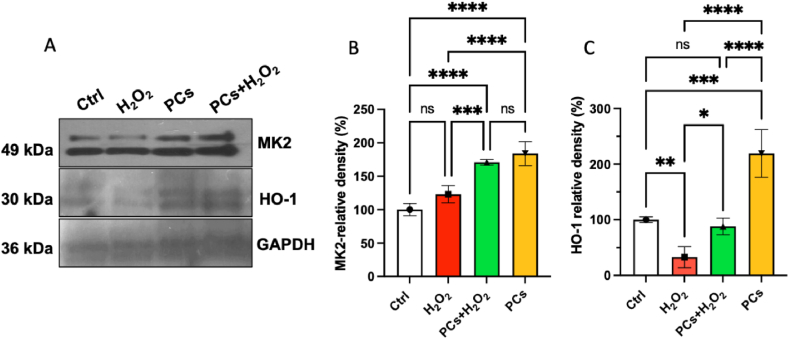
Eﬀect of PCs on the MK2 and HO-1 protein expression in H_2_O_2_-treated HaCaT cells. (A) Representative Western blot of MK2 protein (49 kDa) and HO-1 (30 kDa) of HaCaT cell lysate pretreated with PCs at 100 μg/mL for 12 h prior to treatment with 350 μM of H_2_O_2_ for 30 min. The uncropped version of the Western blot is also available (Fig. S3, Supplementary Information). (B, C) Densitometric analyses of protein levels of MK2 and HO-1. Densitometry values were normalized to the protein loading control, GAPDH. The values are expressed as the mean ± SD of four independent experiments (*n* = 4 per group). Signiﬁcance: one-way ANOVA with Tuckey's multiple comparison test ****p < 0.0001, ***p < 0.001, **p < 0.01 and * *p* < 0.05.

The mitogen-activated protein kinase family (MAPK) is over-activated by external stressors and it plays a relevant role in immune, inﬂammatory, and apoptotic responses [22]. In particular, MK2, a member of MAPK, is known to be involved in oxidative stress [21]. Moreover, a relation between MK2 expression and HO-1 activity has been proposed in hepatobiliary cancer cells [21]. Therefore, the MK2 and HO-1 protein expression was assessed in this work in presence of H_2_O_2_ in HaCaT cells pretreated or not with PCs. Immunoblotting showed that 30 min of H_2_O_2_ treatment decreased the expression of HO-1, while MK2 expression remained unaltered in comparison to the control (Fig. 4A). However, the pretreatment with PCs resulted in a significant up regulation of both MK2 and HO-1 protein expression, when used in co-treatment with H_2_O_2_ in comparison to the H_2_O_2_ treatment alone (Fig. 4A). Indeed, as shown in Fig. 4B, the levels of the band intensity of MK2 range from 32.9 ± 18.0% in the cells treated with H_2_O_2_ to 88.7 ± 15.0% in the co-administration H_2_O_2_/PC pretreated cells. Similarly, the levels of HO-1 protein range from 123.0 ± 13.0% in the HaCaT treated with H_2_O_2_ to 171.0 ± 42.0% in the co-administration H_2_O_2_/PC pretreated cells (Fig. 4C). Interestingly, the levels of MK2 and HO-1 protein expression in the PC pretreated HaCaT cells were significantly greater than the control, reaching the 184.7 ± 18.0% and 219.0 ± 43.0%, respectively (Fig. 4B and C).

### 3.6 Anti-inflammatory activity of hemp PCs

Recently, it has been proposed a novel mechanism of inflammation, where the interaction of oxidative stimulus and inflammatory possess are tightly linked in a positive feed-back manner, which, starting from a non-clinical symptom might chronically lead to either a systemic or local damage [23]. Among the different pathways involved in oxinflammation, NF-κB plays a pivotal role [24]. Moreover, it is now well-established that MK2 is implicated in dampening the activity of NF-κB by regulating p38 [25]. Thus, we thought to evaluate its level upon treatment with PCs in the presence of an inflammatory stimulus. As described previously [26], the assay applied in this work evaluates the ability of either a molecule or a mixture to reduce the NF-κB activation induced by TNFα. The reduction of the luminescence signal is related to the reduction of NF-κB activation.

**Fig. 5:**
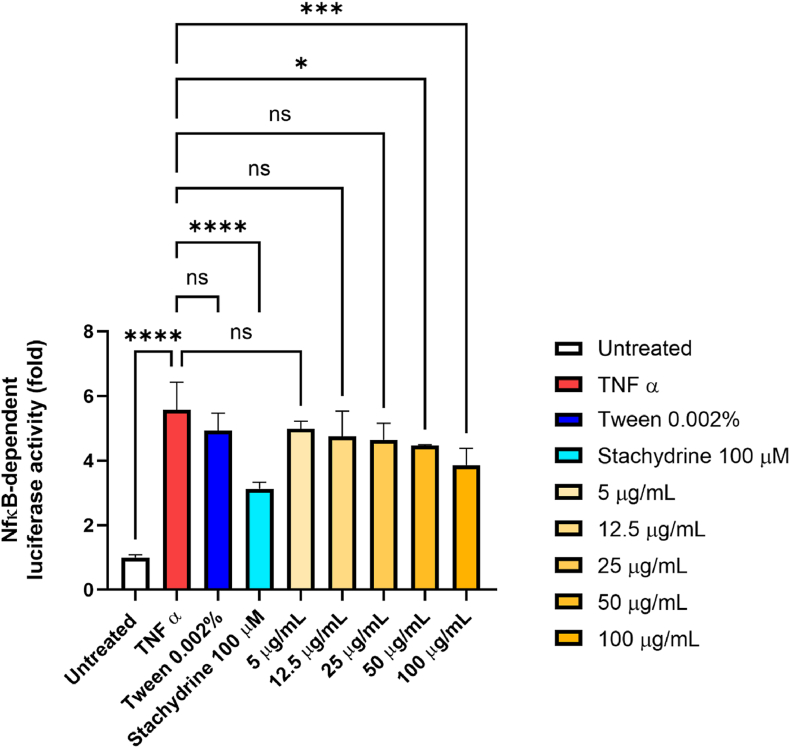
Anti-inflammatory activity of PCs from hemp wax. Values are reported as fold decrease of luciferase signal with respect to cells treated with TNF-α. Dose-dependent activity of hemp wax in a concentration range between 5 and 100 μg/mL and the results from the one-way ANOVA analysis followed by multi-comparison test (****p < 0.0001, ***p < 0.001 and * *p* < 0.05).

As highlighted in Fig. 5, PCs from hemp wax inhibited the upregulation of NF-κB induced by TNFα in a dose-dependent manner (even if at the concentrations 5, 12.5 and 25 μg/mL the reduction is not significant, a trend can be observed). In particular, at the highest concentration tested (100 μg/mL), there is a 1.7-fold reduction, which is not so far from the positive control (stachydrine 100 μM, 2.4-fold). The treatment with both PCs from hemp wax and Tween20 had no significant negative effects on cell viability (Fig. S4, Supplementary Information).

### 3.7 Neutrophil elastase inhibitory activity of hemp PCs

To better characterize the possible anti-inflammatory effects of hemp PCs, the neutrophil elastase activity was assessed. Neutrophil elastase is an important pro-inflammatory mediator that plays a complex role in different physiological and inflammatory processes, such as viral response, oxidative stress [27], liver and pulmonary dysfunctions, cancer [28], as well as in skin chronic inflammations [29]. In addition, differently from other targets, such as peroxisome proliferator activated receptors (PPARs), to the best of our knowledge PCs have never been investigated for their activity on the neutrophil elastase. The ability of hemp PCs to modulate this enzyme was tested using a validated quantitative enzymatic assay on the pro-inflammatory protease. A surprising strong activity of PCs against this target was observed: indeed, as shown in Table 2, the calculated IC_50_ value was 60.6 ± 9.1 μg/mL, whereas, at concentration higher than 100 μg/mL, a plateau of the inhibitory activity >85% was obtained.

**Table 2:** Inhibitory activity of neutrophil elastase exerted by hemp PCs.

Inhibitory activity	Concentration (μg/mL)
<15 % (plateau)	<10
>85 %	100
Linear range	10–75
IC_50_	60.6 ± 9.1

## 4 Conclusions

PCs are so far mainly described as compounds able to interact with lipid metabolism. Although several studies have shown different properties of PCs in the regulation of oxidative stress and inflammation, data regarding their potential activities are still limited concerning the potential molecular targets. Our research, for the first time, clearly showed a significant antioxidant and anti-inflammatory activity elicited by an innovative source of PCs, such as hemp wax. From this matrix, PCs were efficiently extracted and fully characterized by HPLC-ELSD for both the qualitative and quantitative profile. The biologic effect observed might be due to the activation of two separate pathways. The first one involves the increase of MK2 and HO-1 protein. MK2 is involved in the nuclear translocation of NF-E2-related factor (Nrf2), one of the major antioxidant players [30], presumably through Keap-1, while HO-1 acts as an antioxidant protein [31]. The second one is related to the reduction of NF-κB activation and the activation of the neutrophil elastase, both playing an important role in the inflammatory processes.

Even if more research is required to unveil other molecular mechanisms involved in PCs activity, our results suggest that these compounds may have potential for use in the so-called oxinﬂammation processes [23], which, through a long-term oxidative stress, determine a progressive decrease of the physiological adaptive homeostatic response, reinforcing a pro-inﬂammatory status.

## Data availability

The data presented in this study are available on request from the corresponding authors.

## Funding

Financial support for this work was provided by the Italian Ministry of University and Research (MIUR), which provided the PhD grant of Dr. Clarissa Caroli for the PON research project entitled “*Cannabis sativa* L.: a green and sustainable paradigm of medicinal interest”. This study was supported by the FAR2023 Departmental Project entitled “Innovative methods for the extraction, purification and assessment of the bioactivity of policosanols from non-psychoactive *Cannabis sativa* L. biomass in a circular economy perspective” (PI Prof. Federica Pellati). This research was also carried out in the frame of the ALIFAR project, funded by the Italian Ministry of University through the program "Dipartimenti di Eccellenza 2023-2027".

## CRediT authorship contribution statement

**Clarissa Caroli:** Writing – original draft, Methodology, Investigation, Formal analysis, Data curation. **Giovanna Baron:** Writing – original draft, Methodology, Investigation, Formal analysis, Data curation. **Giorgio Cappellucci:** Methodology, Investigation. **Virginia Brighenti:** Writing – original draft, Methodology, Investigation, Formal analysis, Data curation. **Larissa Della Vedova:** Methodology, Investigation. **Francesca Fraulini:** Methodology, Investigation. **Simonetta Oliaro-Bosso:** Writing – review & editing, Visualization. **Andrea Alessandrini:** Methodology, Investigation, Formal analysis, Data curation. **Alfonso Zambon:** Writing – review & editing, Visualization, Data curation. **Gigliola Lusvardi:** Writing – review & editing, Visualization, Data curation. **Giancarlo Aldini:** Writing – review & editing, Visualization. **Marco Biagi:** Writing – review & editing, Visualization, Data curation, Conceptualization. **Lorenzo Corsi:** Writing – review & editing, Visualization, Validation, Supervision, Resources, Project administration, Methodology, Investigation, Funding acquisition, Formal analysis, Data curation, Conceptualization. **Federica Pellati:** Writing – review & editing, Visualization, Validation, Supervision, Resources, Project administration, Methodology, Investigation, Funding acquisition, Formal analysis, Data curation, Conceptualization.

## Declaration of competing interest

Federica Pellati reports financial support was provided by University of Modena and Reggio Emilia, Department of Life Sciences (FAR2023). The other authors have no known competing financial interests or personal relationships that could have appeared to influence the work reported in this paper.
