# Supplementary material for: Extraction, purification and in vitro assessment of the antioxidant and anti-inflammatory activity of policosanols from non-psychoactive Cannabis sativa L
Source: Heliyon. 2024 Apr 26;10(9):e30291. doi: 10.1016/j.heliyon.2024.e30291 (PMC11088244; doi:10.1016/j.heliyon.2024.e30291)
Supplement: Multimedia component 1 [file mmc1.docx]

**Supplementary Information**

**
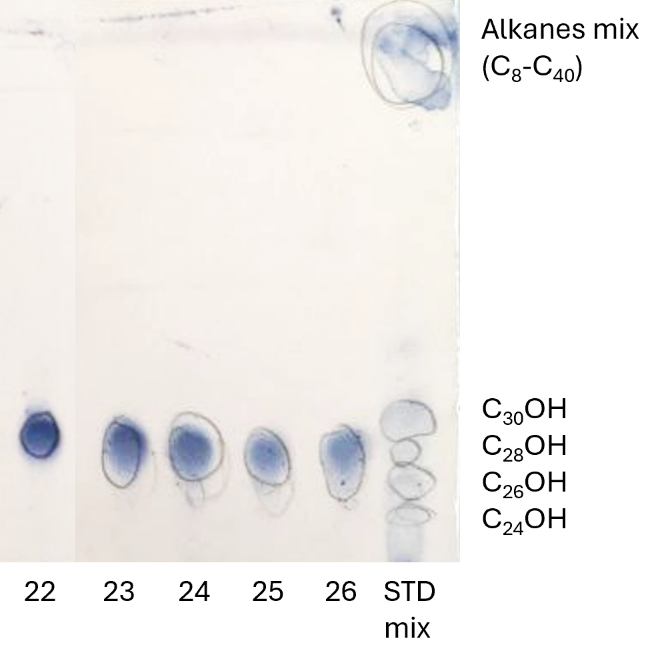
**

**Figure S1:** TLC of fractions 22-26, together with the standard mixture of policosanols and alkanes.

**
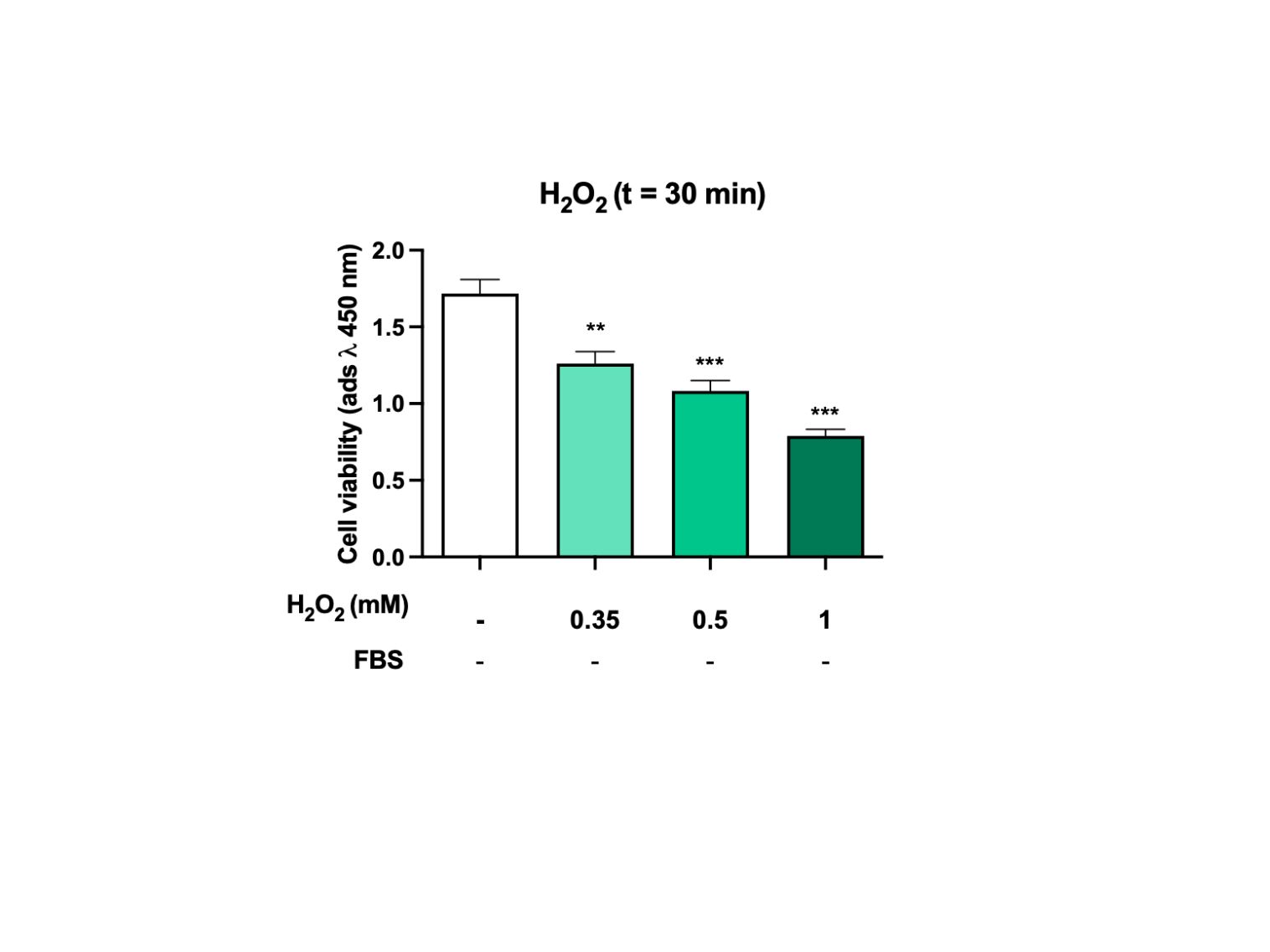
**

**Figure S2:** Cytotoxic effects of H_2_O_2_ on HaCat cell line. Cells were exposed to the indicated concentrations of H_2_O_2_ for 30 min. Cell viability was measured by CCk-8, and values are expressed as the mean ± SD of three independent done in quadruplicate. Significance one-way ANOVA with Tuckey’s multiple comparison test (*** *p* < 0.001 and ** *p* < 0.01 vs Ctrl).


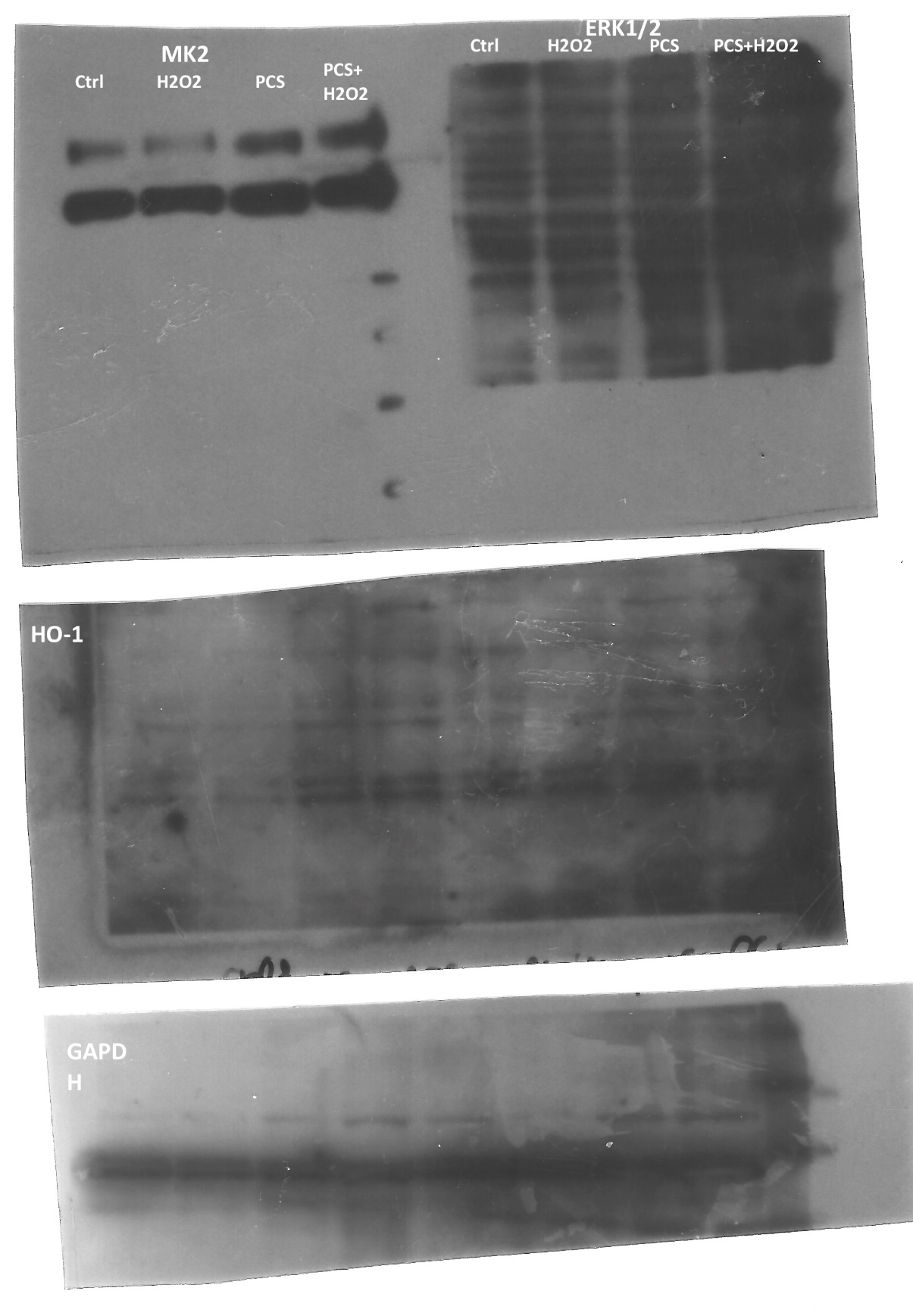


**Figure S3:** Uncropped western blot of MK2 protein (49 kDa) and HO-1 (30 kDa) of HaCaT cell lysate treated with 350 μM H_2_O_2_ for 30 min, PCs at 100 μg/mL for 12 h alone and PCs at 100 μg/mL for 12 h before the treatment with of 350 μM H_2_O_2_ for 30 min**.**


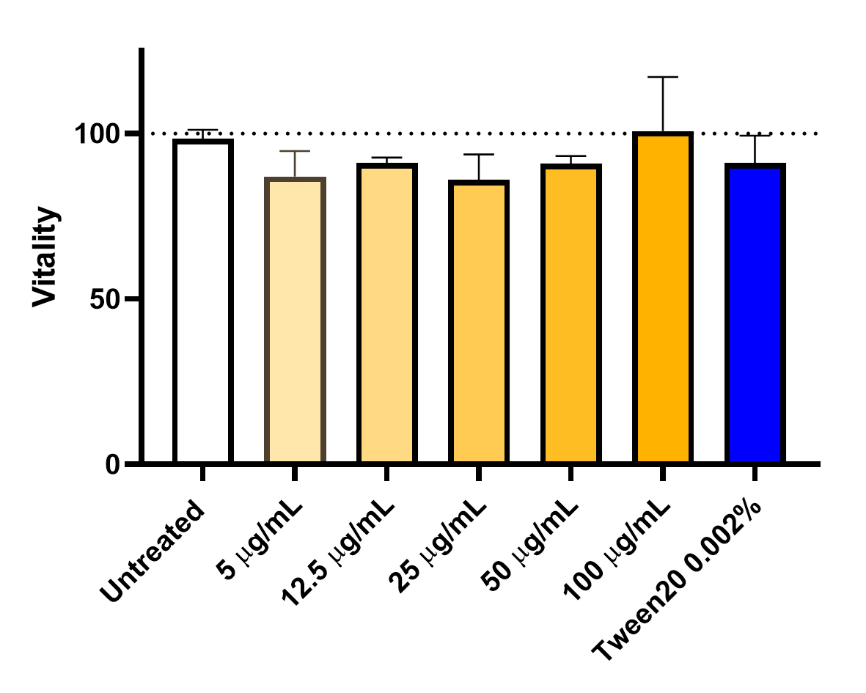


**Figure S4:** Vitality of cells after 18 h pre-treatment with PCs from hemp wax and Tween20 *vs*. untreated cells.
